# Temporal and Spatial Differentiations in Environmental Governance

**DOI:** 10.3390/ijerph15102242

**Published:** 2018-10-12

**Authors:** Benhong Peng, Yue Li, Guo Wei, Ehsan Elahi

**Affiliations:** 1 Research Base of Socialism with Chinese Characteristics Theory System and Science, Nanjing University of Information Science and Technology, Nanjing 210044, China; 2 School of Management Science and Engineering, Nanjing University of Information Science & Technology, Nanjing 210044, China; 3 School of Management, Jiangsu University, Zhenjiang 212001, China; 4 Department of Mathematics & Computer Science, University of North Carolina at Pembroke, Pembroke, NC 28372, USA; guo.wei@uncp.edu; 5 School of Business, Nanjing University of Information Science and Technology, Nanjing 210044, China; ehsanelahi@nuist.edu.cn

**Keywords:** Yangtze River urban agglomeration, environmental governance, DEA model, Malmquist, Tobit model

## Abstract

With the general degradation of environmental carrying capacity in recent years, many developing countries are facing with the dual task of economic development and environmental protection. To explore the issue of urban environmental governance, in this research, we establish a Data Envelopment Analysis (DEA) model to investigate the environmental governance regarding temporal and spatial efficiency. Further, we deconstruct environmental governance efficiency into comprehensive efficiency, pure technical efficiency, and scale efficiency and develop a Tobit model to analyze the influencing factors affecting urban environmental governance efficiency. In addition, the above DEA, Tobit model, and deconstruction of efficiency have been applied to study environmental governance efficiency for the Yangtze River urban agglomeration. Findings include: (1) The gap in environmental governance efficiency between cities is highly noticeable, as the highest efficiency index is 0.934, the lowest is only 0.246, and the comprehensive efficiency index has fallen sharply from 0.708 to 0.493 in the past 10 years; (2) Environmental governance efficiency is basically driven by technological progress, while the scale efficiency change index is the main driver of the technological progress change index; (3) For environmental governance efficiency, urbanization and capital openness are irrelevant factors, economic level and urban construction are unfavorable factors, and industrial structure and population density are favorable factors. These findings will help urban agglomerations to effectively avoid the adverse effects of environmental governance efficiency in economic development, and achieve a coordinated development of urban construction and environmental governance.

## 1. Introduction

Accompanying the accelerated advancement of the global economy, the problem of urban environmental governance has become the focus of all countries. In fact, as early as 1992, the World Organization for Sustainable Development had already targeted the problem by proposing the concept of “environmental efficiency” to measure the environmental costs of urban economic development.

In the context of global attention to environmental issues, achieving environment-friendly economic growth is imperative for many fast developing countries. In China, accompanied by the astonishing development of urban industrialization, numerous urban agglomerations of different sizes have formed so far, such as the Yangtze River urban agglomeration or the Pearl River urban agglomeration. However, under the condition of limited environmental carrying capacity, the environmental problems caused by both the population aggregation and the industrial development are constantly increasing the pressure of environmental governance in urban agglomerations and have become a prominent social issue that affects the production and life of urban residents.

Consequently, studying the environmental governance of urban agglomeration is an important exploration of environmental issues in the process of urbanization. The analysis of the environmental conditions of the Yangtze River, which is the longest river system in the country, crossing 31 provinces, is of great significance to other urban agglomerations facing the same problems.

At present, research on environmental governance at home and abroad is mainly focused on:

(1) Environmental Governance Efficiency (EGE)

Gan and Wang [1] (2013) used the Data Envelopment Analysis (DEA) model, and Malmquist index to calculate the EGE and dynamic change of the Yangtze River Delta cities, a coastal area in eastern China, 51,000 square kilometers that includes Nanjing, Zhenjiang, Yangzhou, Taizhou, Changzhou, Wuxi, Suzhou, Nantong, with a population of 50 million. They found that the EGE appeared generally higher and fluctuating upward in the Yangtze River Delta.

Zaim O [2] (2000) used nonparametric techniques to measure the EGE of Organisation for Economic Co-operation and Development, formulated an EGE index, and compared pollution control across countries. The results showed that strict limit on carbon emissions will lead to output losses. Mandal, Madheswaran [3] (2007) used DEA to measure the EGE of India in a joint production framework. The results showed that the cement industry may expand the desired production and shrink the unfavorable output in the face of environmental regulation. Van Leeuwen, Koop, and Sjerps [4] (2016) made a baseline assessment of water resource governance in Europe and found that cities in transitional and developing countries are prone to water risk. Tang, Zhang, and Liu [5] (2016) used the slackness measurement (SBM) poor model to analyze the efficiency of environmental regulation. The results showed that environmental regulation efficiency presented a “polarization” effect and the environmental regulation total factor productivity had a clear “aggregation” effect in China.

(2) Environmental governance research methods

Reinhard et al. [6] (2000) used the Stochastic Frontier Approach and Data Envelopment Analysis (DEA) to estimate the comprehensive EGE measures and compared the two methods. The results showed that DEA is more suitable for calculating the EGE scores of all specifications. Luo [7] (2012) proposed an EGE evaluation method based on slack variables, which was applied to the efficiency of industrial environment in China. It was found that about two-thirds of the decision-making units were not effective. Tang et al. [8] (2012) constructed a low carbon manufacturing evaluation index system and proposed evidence for industrial policy innovation based on this evaluation system. Tu and Qiu [9] (2013) based their research on the DEA-based directional distance function method to examine China’s industrial EGE and found that environmental technology efficiency assessed by traditional techniques underestimated the EGE. Kuosmanen and Entertainer [10] (2015) evaluated how Data Envelopment Analysis aggregated environmental pressures into a single environmental damage index by assessing the EGE of urban road transport in eastern Finland. Koop [11] (2017) analyzed the water, waste, and climate change challenges of cities, and used an empirical method to achieve consistent comparisons between cities and then proposed governance options.

(3) Environmental governance policy

Gunningham [12] (2009) examined the relationship between old and new governance from the perspective of three Australian case studies, and revealed the importance of the state in the “shadow of hierarchy”. Tang [13] (2012) pointed out the challenges faced by China’s low-carbon manufacturing industry and proposed related countermeasures through comparison of China’s energy consumption with other countries. Bruce and William [14] (2014) reviewed the dynamic change in China’s local environmental governance and proposed solutions to the problems of “policy implementation gap” and “participation gap”. Kitagawa, Geng [15] (2017) has studied China’s environmental governance policy pipeline since 1979, and believes that implementing environmental governance policies should be centered on local governments. From the four aspects of legal, management, governance mechanism, and policy, Tang [16] (2017) combed the refined governance system formed by Japan in the process of environmental governance, and proposed the improvement path of China’s environmental governance. The above scholars’ research methods are relatively simple, failing to fully reflect the status of efficiency development. The area of research objects is relatively extensive; the research on urban agglomerations as an object and urban EGE data as indicators is not sufficient, and the study of EGE in large regions does not reflect the heterogeneity of regional development. Furthermore, it lacks of space-time analysis of efficiency differences and the study of the trend of efficiency development.

This article uses data from 8 cities of the Yangtze River urban agglomeration from 2007 to 2016 to build a DEA model, for calculating the efficiency of environmental management. On one hand, it comprehensively analyzes the environmental governance of the Yangtze River urban agglomeration; by contrast, it also deconstructs the EGE to compare and analyze the spatial and temporal differences in EGE among cities, and further discusses relevant factors affecting the EGE of the Yangtze River urban agglomeration, expecting to fill the gap in space-time differentiation study and to provide useful ideas for the governance of urban agglomerations.

In the remainder of this article, the contents are arranged in the following way: Section 2 introduces the DEA model and data sources, and establishes an EGE indicator system. Section 3 analyzes the difference of EGE between cities within the Yangtze River urban agglomeration from the perspective of time and space, and uses the Malmquist exponential decomposition to investigate the impact of various indicators on EGE. Section 4 uses the Tobit regression model to analyze the magnitude and impact of important factors affecting the EGE of cities in the Yangtze River urban agglomeration. In Section 5, we summarize the environmental management efficiency of the Yangtze River urban agglomeration and give relevant suggestions.

## 2. Research Methodology

In this section, we study the rationality of the data envelopment analysis (DEA) method for our problem and examine the data sources. Subsequently, we construct an EGE index system based on the ecological status regarding the Yangtze River urban agglomeration for exploring influencing factors of EGE.

### 2.1. DEA Model

DEA, or data envelopment analysis, is a research method proposed by well-known American researchers Charnes, Copper, and Rhodes in 1978 to evaluate efficiency among samples [17] (Charnes, Cooper, Rhodes, 1982). When using the DEA model for efficiency calculation, an indicator system and a decision unit [18,19] (DU) (Fare and Grosskopf, 1986; Huppes and Ishikawa, 1986) (Decision Making Units, DMU) should be established: If efficiency is 1, the output of the DU is on the frontier boundary, which indicates that the input is effective; if efficiency is less than 1, the output of the DU is at a distance from the frontier boundary, which means that there is still some distance between the effect of investment and the ideal result. Specifically, DEA is a model that uses mathematics planning to establish evaluations, determines relatively effective production frontiers, and projects each DU onto the production frontier. The relative effectiveness of each DU is compared by the distance from the DU to the production frontier [20] (Wang, et al., 2010). The specific model formula is as follows:

(1) $$\begin{array}{c} \rho^{*}=Min\frac{1-\frac{1}{m}\sum_{i=1}^{m}\frac{{\bar{s}}_{r}}{x_{io}}}{1+\frac{1}{s_{1}+s_{2}}\left(\sum_{r=1}^{x_{1}}\frac{{s^{g}}_{r}}{{y^{g}}_{ro}}+\sum_{r=1}^{x_{2}}\frac{{s^{b}}_{r}}{{y^{b}}_{ro}}\right)},\\ x_{o}=X\lambda +\bar{s},\\ y^{g}{}_{o}=Y^{g}\lambda -s^{g},\\ y^{b}{}_{o}=Y^{b}\lambda +s^{b},\\ \bar{s}\geq 0,s^{g}\geq 0,s^{b}\geq 0,\lambda \geq 0 \end{array}$$

where *x* is a single input to the production unit, *X* is the input vector of the production unit, m is the quantity of production inputs *x*, *y* is a single output, and *Y* is the output vector of the production unit. $\rho^{*}$ is the efficiency value, $\bar{S}$ stands for the amount of excess redundancy, $S_{r}^{g}$ means that the expected output is insufficient, $S_{r}^{b}$ stands for an excess surplus of an undesired output, and λ denotes the weight of input and output.

When $\rho^{*}=1$ and $\bar{S}=0$, or $S_{r}^{g}=0$, or $S_{r}^{b}=0$, the DMU is an effective unit of EGE.

When $\rho^{*}=1$ and $\bar{S}\neq 0$, or $S_{r}^{g}\neq 0$, or $S_{r}^{b}\neq 0$, it is a weakly effective unit, that is, while it reduces the input of redundant production unit $\bar{S}$, the output of $y_{0}$ does not change.

When $0\leq \rho^{*}<1$, it is an invalid unit of EGE, that is, reducing the original input $\rho^{*}$ can leave the production unit $y_{0}$ output unchanged. If the DMU is improved to be an effective unit, it requires some improvements in input, expectation, and unintended output.

### 2.2. Data Sources and Indicators Selection

The data used in this paper come from Jiangsu Environmental Statistics Annual Report (2008–2017), Environmental Status Bulletin (2007–2016), Environmental Statistical Yearbook (2008–2017), and Statistical Yearbook (2008–2017). We selected 80 pairs of environment-related data from 2007 to 2016 for the DEA model, and 5 indicators from each set of data.

At present, urban environmental governance is dominated by the governments in different administrative regions, and environmental governance in each region is relatively independent. Therefore, the environmental governance process in each administrative region of the Yangtze River urban agglomeration can be regarded as a DU (Fare R, 1986). The environmental governance economic investment is the input of DMU. The EGE is taken as the output of DMU. The EGE is determined by the intended output and unintended output, both caused by the input of environmental governance. Since DEA theory requires that the number of indicators between input and output is less than or equal to half the total number of DMUs in the DU [21] (Guo and Zheng, 2009), we selected the environmental capital investment as the input variable, GDP as the expected output index, and the regional emissions, waste water discharge, and unprocessed industrial solid waste as unintended output indicators, for the following considerations:

1. They have sufficient literature support.

References Gai et al. (2014), Li Min (2017), Wu H Q et al. (2014) used these five indicators to construct an evaluation system for environmental governance efficiency, which verified the rationality of these five indicators.

2. They are representative.

Capital investment is the most important investment in environmental protection, and economic level is an important indicator of efficiency. Waste water and solid waste are the main sources of environmental pollution. Therefore, we used GDP as the expected output, and used the waste water, waste gas, and solid waste as undesired outputs.

Subsequently, a three-level, three-dimensional index evaluation system was formed (Table 1). At the same time, the urbanization level, industrial structure, economic development level, urban construction, population density, and capital openness index were selected to study the influencing factors of EGE for the Yangtze River urban agglomeration. The descriptive statistical characteristics of input and output are shown in Table 2.

## 3. EGE for the Yangtze River Urban Agglomeration

To grasp the current status of EGE for the Yangtze River urban agglomeration and the impact of various indicators on EGE, we used DEAP 2.1 (University of Queensland, Brisbane, Australia) and OpenGeoDa software tools (University of Chicago, Chicago, IL, USA) to analyze the spatial differences and dynamic evolution of the Yangtze River urban agglomeration from the perspective of time and space, and then the Malmquist Index Decomposition was employed to explore the relationship between each index and the EGE.

### 3.1. The Temporal and Spatial Analysis of EGE for the Yangtze River Urban Agglomeration

Before analyzing the spatial and temporal differentiation of the environmental management efficiency for the Yangtze River urban agglomeration, we started with an overall analysis of EGE.

#### 3.1.1. Overview of the EGE

According to the results produced from DEAP 2.1, as shown in Table 3, the overall EGE of the cities in the Yangtze River urban agglomeration is low. In terms of average values, the EGE of Nanjing, Changzhou, Wuxi, and Zhenjiang is relatively high, with the efficiency indices being 0.934, 0.849, 0.742, and 0.698, respectively, while the EGE for Yangzhou, Suzhou, Taizhou, and Nantong is relatively low, with the efficiency indices of 0.423, 0.371, 0.353, and 0.264, respectively. This shows a maximum difference of 0.67 in the index, between Nanjing (0.934) and Nantong (0.264), which implies that the EGE of the Yangtze River urban agglomeration is very different by location, and this is not contributive to the co-development of urban agglomerations.

#### 3.1.2. Research on Time Differentiation of EGE

**Figure 1:**
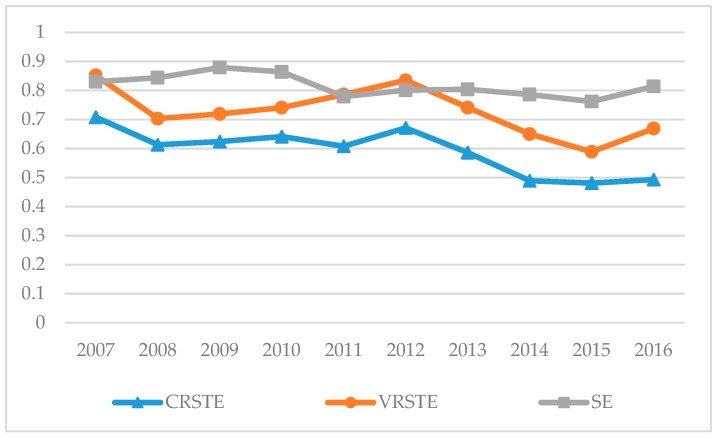
Decomposition of EGE in the Yangtze River urban agglomeration.

The EGE is deconstructed into three parts: Comprehensive efficiency (CRSTE), pure technical efficiency (VRSTE), and scale efficiency (SE). CRESTE (comprehensive efficiency) is used to measure whether environmental governance meets the overall requirements and achieves maximum economic and social benefits; VRSTE (pure technical efficiency) represents the maximum output that a decision-making unit can achieve under a given input. It is used to measure the magnitude of the effect on the output unit due to management and technology factors; SE (scale efficiency) examines whether the decision-making units are operating at the most appropriate scale of investment under certain conditions of technology. By calculating CRSTE, we can know the overall environmental governance efficiency of Jiangsu Province. By calculating VRSTE and SE, we can judge the influence of technology and scale on the governance efficiency in the environmental governance process of Jiangsu Province. The average data of the three parts from 2007 to 2016 are selected for differential analysis (Table 4), and Figure 1 is plotted to analyze the overall temporal efficiency of the Yangtze River urban agglomeration. The study finds that the overall EGE of the Yangtze River urban agglomeration is in the upper middle level, and there are problems of excessive resource input or serious shortage of output. In 2007, the EGE reached a maximum, and afterwards the efficiency index gradually decreased. From 2012, the EGE index began to decrease rapidly. The decline indicates that a rapid development of the city’s economic level may have caused some negative impact on the environment. In addition, the SE (0.822) is larger than the VRSTE (0.76), which in turn is larger than the CRSTE (0.6), and pure technological efficiency is similar to the curve of changes in CRSTE.

#### 3.1.3. Research on Spatial Differentiation of EGE

**Figure 2:**
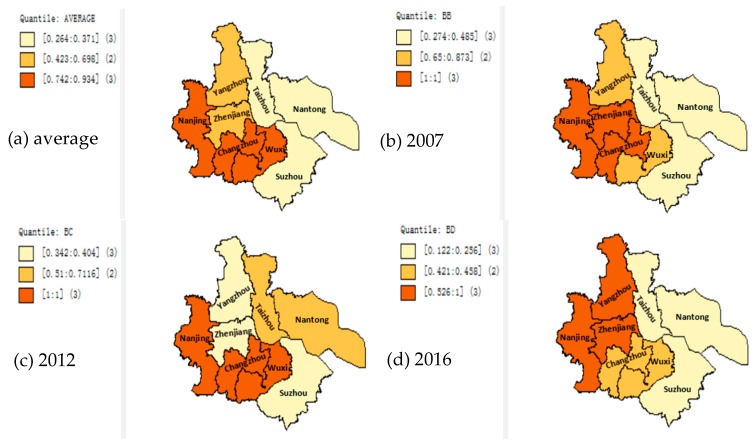
Distribution of EGE of the Yangtze River urban agglomeration. (**a**) Distribution of the average efficiency of the cities over 10 years; (**b**) Efficiency distribution of cities in 2007; (**c**) Efficiency distribution of cities in 2012; (**d**) Efficiency distribution of cities in 2016.

Using OpenGeoDa software [25] (Tang and Zhang, 2008), take the average value of the EGE of the Yangtze River urban agglomeration from 2007 to 2016 as the reference, divide the efficiency value into three categories and plot in Figure 2a; Choose the EGE of the Yangtze River urban agglomeration in 2007, 2012, and 2016, respectively, as the reference, plot in Figure 2b–d. By analyzing the result of grade distribution (Figure 2), the average efficiency values in Nanjing, Changzhou, and Wuxi are from 0.742 to 0.934, which are of high efficiency levels. The average value of efficiency in Yangzhou and Zhenjiang is between 0.423 and 0.698, which is a typical efficiency level; the average efficiency of Taizhou, Nantong, and Suzhou is between 0.264 and 0.371, which is an inefficient level. In 2007, the EGE of the Yangtze River urban agglomerations was generally higher, with the indices of Nanjing, Zhenjiang, and Changzhou reaching 1. This indicates that in 2007, the level of economic development was not high and the environmental pollution of each city was not serious. As a result, environmental governance is relatively effective. By 2012, Nantong and Taizhou were transformed from low efficiency to high efficiency, indicating that environmental management was emphasized in the process of urban development, and the EGE was improved, while Yangzhou and Zhenjiang became inefficient regions, indicating that the environmental pollution control was neglected during urban economic development process. In addition to Nanjing, Zhenjiang, and Yangzhou, in 2016, the efficiency of environmental management in other cities was lower than the average value, indicating that the Yangtze River urban agglomeration did not maintain the simultaneous development of environmental governance and economy in the process of economic development. The EGE of Nanjing has been at a relatively high level, indicating that its input and output have reached an optimized level; Yangzhou and Zhenjiang were promoted to high-efficiency areas in 2016, indicating that it may take the old pattern of “polluting first and cleaning up later”, and environmental governance did not advance in a coordinated manner with urban construction. From a spatial perspective, the level of urban development in southern Jiangsu is relatively fast, and the EGE is also high; by contrast, the level of urban development in central Jiangsu is relatively slow and the EGE is relatively low.

### 3.2. Malmquist Analysis of EGE in the Yangtze River Urban Agglomeration

Malmquist [26] (Malmquist, S. 1953) exponential decomposition is to decompose the efficiency change index (effch) into a technical efficiency change index (techch) and technological progress index (tfpch), and then decompose techch into a pure techch index (ptech) and scale efficiency change index (seffch). Ptech reflects the impact of technological level on productivity; scale efficiency change reflects the impact of scale economy on productivity and the proximity of DMU to the optimal scale; technological change reflects the impact of moving production frontiers on productivity. If effch, ptech, seffch, and techch are greater than one, this indicates that this indicator has a positive effect on EGE; if it is less than 1, the opposite is true.

Through the analysis of the decomposition results (Table 5), the effch of Nanjing and Zhenjiang were greater than 1, which were 1.204 and 1.098 respectively, indicating that both cities have made progress in urban environmental governance. These two cities have increased by 20.4% and 9.8% respectively, mainly driven by technological progress, indicating that Nanjing and Zhenjiang have achieved good results in environmental governance technology over the past 10 years. The effch of the other six cities were all less than 1, which were 0.961 in Yangzhou, 0.921 in Taizhou, 0.924 in Changzhou, 0.989 in Wuxi, 0.896 in Suzhou, and 0.952 in Nantong, and the degree of regression in environmental governance was 1.1% to 10.4%. Among them, Suzhou City had the most obvious step backwards for more than 10%, mainly due to techch and seffch. This shows that although Suzhou is a major city for economic development in our province, it has not improved its environmental governance technical efficiency, and its economic scale has not developed rapidly. In order to harmonize environmental and economic development, Suzhou must pay attention to economic scale and technological efficiency. The decline of EGE in other 5 cities is not obvious. The main influencing factor is the level of technical efficiency.

**Figure 3:**
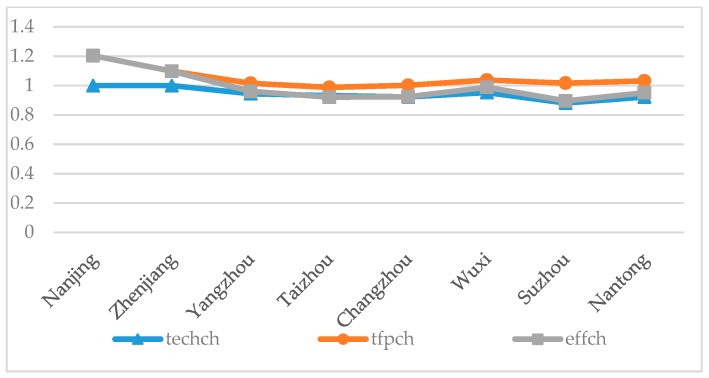
Efficiency change index and index decomposition of EGE of the Yangtze River urban agglomeration in 2007–2016.

From Figure 3, we found that technological progress is the main factor driving the improvement of the EGE; Cities with high tfpch universally have high EGE; Suzhou City is more special, as the city’s tfpch is higher than others, but the EGE is lower, indicating that the degree of technological progress in Suzhou is lower than the degree of backwardness in technical efficiency and scale efficiency.

**Figure 4:**
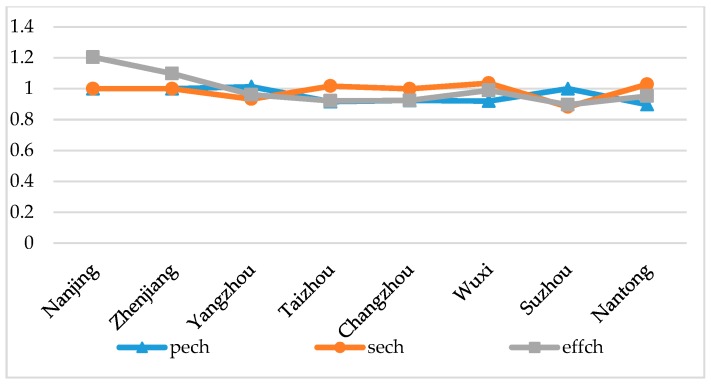
Index of efficiency change and index decomposition of EGE of the Yangtze River urban agglomeration in 2007–2016.

Through the analysis of technical progress index decomposition results (Figure 4), we found that, except for the seffch of Yangzhou, Changzhou, and Suzhou, which is lower than 1, the other cities are all greater than 1 and the ptech of each city is basically lower than 1. This shows that scale efficiency change is the main factor that drives technological progress. Therefore, the improvement of urban EGE must make urban development closer to the optimal scale.

## 4. Influencing Factors of EGE

After obtaining the calculation results from the DEA model, it was found that the EGE of the Yangtze River urban agglomeration is also affected by other factors, in addition to the output indicators. Therefore, we selected other indicators as the main influencing factors of EGE and analyzed the effects of these influencing factors on EGE using the Tobit regression model.

### 4.1. Main Influencing Factors of EGE

The EGE of the Yangtze River urban agglomeration, which was previously calculated using the DEA model, is also affected by other factors than the output indicators of Malmquist model. To further analyze the influencing factors of EGE, the aspects listed below are selected as the main influencing factors of EGE on the basis of development status of the Yangtze River urban agglomeration (Table 6):

### 4.2. Tobit Regression Study

The Tobit regression model can be used to solve regressions with restricted conditions. The regression equation is given as follows [30,31] (Tobin, 1956; Coelli, 1998):

(2) $$\begin{array}{c} y=\beta^{\prime }xi+ui,\\ {y^{*}}_{i}=y_{i},\;if\;{y^{*}}_{i}>0,\\ {y^{*}}_{i}=0,\;if\;{y^{*}}_{i}\leq 0. \end{array}$$

In the Equation, $y_{i}^{*}$ is a potential variable, observed when the latent variable is greater than 0, and the value is $y_{i}$, truncate at 0 when it is less than or equal to 0; $X_{i}$ is an independent vector, β is a coefficient vector, the error term $u_{i}$ is independent and obeys the normal distribution: $u_{i}\sim N\left(0,\sigma^{2}\right)$.

A metrology analysis software Eviews (Microsoft, Redmond, WA, USA) was used to make a unit root test; the indicators were brought into Eviews for Tobit regression (Table 7).

The Tobit regression results indicate that the significance of the urbanization level and capital openness has not passed the test, which is to say that its effect on the EGE is not very obvious. Therefore, removing both of them for Tobit regression again, we have obtained the following results (Table 8).

The economic development level and urban construction have an adverse effect on the EGE at the significant level of 10% and 1%. For every 1% increase in per capita GDP, the EGE drops by 3.05 × 10^−6^%; for every 1% increase in investment in infrastructure construction, the EGE will decline by 9.496233%. Industrial structure and population density have a favorable effect on the EGE at the significant level of 5% and 10%, respectively. For every 1% boost in the GDP of the tertiary industry, the EGE will increase by 2.208085%; for each 1% increase in population density, the EGE will increase by 6.52 × 10^−5^%.

### 4.3. Analysis and Discussion of Results

The economic development level and urban construction have an adverse effect on the EGE. However, the overall income level of the Yangtze River urban agglomeration is at the top of the country, but the EGE is at the middle-to-upper level. Although the income level increases, the ability to generate pollutants also increases, and the generated domestic garbage has not been properly disposed of, resulting in a decrease of urban EGE. That is, the ability to produce pollution has increased, but the awareness of protecting the urban environment has not been strengthened. With the increase of the construction for urban infrastructure, more pollutants will be generated and more resources will be needed to deal with them, which will inevitably reduce the urban EGE.

The industrial structure has a beneficial effect on the urban EGE. As an important developing area in Jiangsu province, the Yangtze River urban agglomeration has always been dominated by the tertiary industry. Suzhou and Nanjing are high-tech cities. Wuxi and Nantong mainly develop the textile industry. Therefore, vigorous improvement of the tertiary industry is conducive to the EGE. Additionally, the service industries, such as scientific research design, logistics industry, and financial industry in the tertiary industry, contribute to the positive effect of productivity and EGE. However, the low EGE in Suzhou is primarily affected by excessively invested on but inadequately utilized resources in environmental protection.

Population density has a beneficial effect on the urban EGE. Nanjing is the most obvious example. More and more laborers in other provinces are pouring into Nanjing, which greatly increases the population density of Nanjing. However, the EGE of Nanjing is the highest in the Yangtze River urban agglomeration. The reason is that the population density has brought about the improvement of living standards and education. The increase in education level, economic growth, and awareness of environmental protection should be greater than the environmental pressure. Therefore, large immigration numbers are in fact conducive to the improvement of urban EGE rather than having a negative effect.

The level of urbanization and the capital openness have no significant effect on the EGE of the Yangtze River urban agglomeration. This shows that in improving urban environmental governance measures, the level of urbanization and capital openness are not the main factors to consider.

## 5. Conclusions

### 5.1. Conclusions and Suggestions

In general, the EGE of the Yangtze River urban agglomeration is relatively low. There are 4 cities with relatively high efficiency, namely Nanjing, Changzhou, Wuxi, and Zhenjiang, of which Nanjing has the highest efficiency, as high as 0.934; 4 cities have relatively low efficiency, namely Yangzhou, Suzhou, Taizhou, and Nantong, of which Nantong has the lowest efficiency, as low as 0.246.

Judging from the time dimension, the overall EGE of the Yangtze River urban agglomeration is in the upper middle level. In 2007, the EGE reached its maximum value, and then the index gradually decreased. Since 2012, the EGE index has rapidly declined. In the course of economic development, we should improve the environmental management agencies and strengthen the management and supervision of urban environmental governance to ensure that the EGE is gradually improved, instead of promoting the development of urban economy at the expense of the environment.

Judging from the spatial perspective, the urban development in the southern part of Jiangsu is relatively fast, and the EGE is also high. The urban development in northern Jiangsu is relatively lagging behind, causing greater damage to the environment and a lower EGE. Therefore, it is necessary to construct the exchange system of the Yangtze River urban agglomeration with a shared concept, in order to achieve interconnection and interoperability among the eight cities, and to promote environmental governance in coordination.

According to the DEA-Malmquist index decomposition analysis, the EGE of the Yangtze River urban agglomeration is basically driven by tfpch, and the seffch is the main driver of tfpch. Suzhou and Nantong have invested much more in environmental governance than other cities, but the EGE is the last. The main reason is that environmental governance technology is not mature and capital investment has little effect. Therefore, it is particularly important to accelerate the development of science and technology. The Yangtze River urban agglomeration needs to improve technological innovation capabilities; accelerate the elimination of backward and immature environmental technologies; and communicate actively with other environmentally more efficient regions and introduce advanced environmental protection technologies.

From the Tobit regression research analysis, it is concluded that urbanization level and capital openness have no significant impact on urban EGE; the economic development level and urban construction have an adverse impact on urban EGE; and industrial structure and population density have a beneficial impact on urban EGE. Therefore, optimizing industrial structure during the development of the Yangtze River urban agglomeration has a positive effect on improving regional environmental quality. Transferring energy-intensive and highly-polluting enterprises to knowledge-intensive and environment-friendly industries can enhance the harmonious advancement of the economy and environment. In addition, the population density has a beneficial effect on the efficiency of urban environmental governance. Therefore, the government should encourage the public to join the environmental management, through the three-party supervision of the government, enterprises, and the public to promote the improvement of urban EGE.

### 5.2. Study Limitations and Prospects

Although we tried to select influencing factors comprehensively and systematically, it is inevitable to have some missed considerations and it is difficult to cover all the factors affecting the EGE. In addition, we have only studied the efficiency of China’s interprovincial environmental governance from a macro perspective. Due to the lack of more specific industry characteristics, it is difficult to provide targeted policy recommendations for different industries. Therefore, the next step is to study the EGE of various industries, to explore the mechanism of environmental governance-influencing factors from a micro perspective, to provide a deeper industry foundation for China’s EGE, and thus to formulate more practical policy guidance for microproducers.

## Figures and Tables

**Table 1:** Indicator system for Environmental Governance Efficiency (EGE) of the Yangtze River urban agglomeration.

Comprehensive Layer		Target Layer	Indicator Layer	Literature Basis
Yangtze River City Group EGE Index	Input indicators	Capital investment	Total investment in environmental protection	Gai et al. [22] (2014) Li Min [23] (2017) Wu H Q et al. [24] (2014)
	Output indicators	Expected output	GDP	
		Undesired output	Exhaust gas emissions	
			Wastewater discharge	
			Solid waste untreated	

**Table 2:** Descriptive statistical characteristics of input-output variables in 2007–2016.

Variable Type	Specific Variable	Unit	Maximum Value	Minimum Value	Average Value	Standard Deviation
Input indicator	Total investment	108 yuan	641.23	10.12	116.33	130.63
Output indicator	GDP	108 yuan	15475.09	1201.82	5027.89	3272.20
	Exhaust gas emissions	108 cubic meter	15161.3	0.24	46.78	3544.85
	Waste water discharge	108 ton	7.46	0.56	2.53	1.75
	Solid waste untreated	104 ton	501.85	839	4122.76	74.34

**Table 3:** Results of EGE for the Yangtze River urban agglomeration from 2007 to 2016.

DMU	2007	2008	2009	2010	2011	2012	2013	2014	2015	2016	Average
**Nanjing**	1.00	0.84	1.00	1.00	0.79	1.00	0.70	1.00	1.00	1.00	0.93
**Zhenjiang**	1.00	0.66	0.49	0.62	0.30	0.34	1.00	0.79	0.75	1.00	0.69
**Yangzhou**	0.87	0.29	0.44	0.38	0.69	0.39	0.24	0.21	0.15	0.52	0.42
**Taizhou**	0.48	0.36	0.32	0.33	0.54	0.51	0.28	0.23	0.17	0.25	0.35
**Changzhou**	1.00	1.00	1.00	0.44	1.00	1.00	1.00	0.88	0.70	0.45	0.84
**Wuxi**	0.65	0.59	1.00	1.00	1.00	1.00	1.00	0.44	0.31	0.42	0.74
**Suzhou**	0.38	1.00	0.41	0.45	0.34	0.40	0.24	0.20	0.14	0.12	0.37
**Nantong**	0.27	0.19	0.31	0.32	0.24	0.71	0.20	0.13	0.09	0.13	0.26

**Table 4:** The average of comprehensive efficiency (CRSTE), pure technical efficiency (VRSTE), and scale efficiency (SE) of the environmental management in the Yangtze River urban agglomeration from 2007 to 2016.

Year	CRSTE	VRSTE	SE
**2007**	0.70	0.85	0.83
**2008**	0.61	0.70	0.84
**2009**	0.62	0.71	0.87
**2010**	0.64	0.74	0.86
**2011**	0.60	0.78	0.77
**2012**	0.67	0.83	0.80
**2013**	0.58	0.74	0.80
**2014**	0.48	0.65	0.78
**2015**	0.48	0.58	0.76
**2016**	0.49	0.66	0.81

**Table 5:** Malmquist exponential decomposition results of EGE of the Yangtze River urban agglomeration from 2007 to 2016.

DMU	effch	techch	tfpch	ptech	seffch
**Nanjing**	1.20	1.00	1.20	1.00	1.00
**Zhenjiang**	1.09	1.00	1.09	1.00	1.00
**Yangzhou**	0.96	0.94	1.01	1.01	0.93
**Taizhou**	0.92	0.93	0.98	0.91	1.01
**Changzhou**	0.92	0.92	1.00	0.92	0.99
**Wuxi**	0.98	0.95	1.03	0.92	1.03
**Suzhou**	0.89	0.88	1.01	1.00	0.88
**Nantong**	0.95	0.92	1.03	0.89	1.02
**mean**	0.98	0.94	1.04	0.95	0.98

Note: DMU refers to research decision unit (DU); effch refers to efficiency change index; techch refers to technological efficiency change index; ptech refers to pure technical efficiency change index; seffch refers to scale efficiency change index; tfpch refers to technological progress index.

**Table 6:** Factors affecting the EGE of the Yangtze River urban agglomeration.

Explanatory Variable	Definitions and Units	Shorthand	Literature Basis
The level of urbanization	Proportion of urban population (%)	X1	Gai Mei et al. (2012) Wang Qin et al. [27] (2014) Wang Qing [28] (2015) Song Yanan [29] (2012)
Industrial structure	Tertiary industry share of GDP (%)	X2	
Economic level	Per capita GDP (¥)	X3	
Urban construction	Infrastructure investment (100 million ¥)	X4	
Population density	Population density (person/ sq.km.)	X5	
Capital openness	Actual use of foreign investment (10,000 $)	X6	

**Table 7:** Results of Tobit regression analysis on EGE influencing factors (1).

Variable	Coefficient	Std. Error	Z-Statistic	Prob.
**SER02**	−0.58	0.48	−1.21	0.22
**SER03**	4.19 ***	1.61	2.59	0.01
**SER04**	−5.77 × 10^−6^ **	2.30E−06	−2.51	0.01
**SER05**	−10.78 ***	2.52	−4.26	0.00
**SER06**	7.14 × 10^−5^ *	4.19E−05	1.70	0.08
**SER07**	2.63 × 10^−7^	1.96E−07	1.34	0.18
**C**	−0.38	0.37	−1.01	0.30

Note: SER01 = EGE, SER02 = X1, SER03 = X2, SER04 = X3, SER05 = X4, SER06 = X5, SER07 = X6. * 10% of significance level, ** 5% of significance level, *** 1% of significance level.

**Table 8:** Results of Tobit regression analysis of influencing factors (2).

Variable	Coefficient	Std. Error	Z-Statistic	Prob.
**SER03**	2.20 **	0.94	2.34	0.01
**SER04**	−3.05 × 10^−6^ *	1.66E−06	−1.8350	0.06
**SER05**	−9.4962 ***	2.27	−4.18	0.00
**SER06**	6.52 × 10^−5^ *	3.79E−05	1.71	0.08
**C**	−0.03	0.31	−0.09	0.92

Note: SER01 refers to EGE; SER03 refers to factor X2; SER04 refers to factor X3; SER05 refers to factor X4; SER06 refers to factor X5. * 10% of significance level; ** 5% of significance level; *** 1% of significance level.
